# Isolated necrotizing scleritis revealing silent pulmonary granulomatosis with polyangiitis: A case report of rare diagnostic clue

**DOI:** 10.1097/MD.0000000000051271

**Published:** 2026-10-23

**Authors:** Nabaa Ihsan Awadh, Faiq I. Gorial, Ahmed Dheyaa Al-Obaidi, Mohammed Alghannami, Karam Fareed Nawara, Marafi Jammaa Ahmed, Aya Ahmed Shimal, Ibtisam Hussein Al Obaidi

**Affiliations:** a Rheumatology Unit, Department of Medicine, College of Medicine, University of Baghdad, Baghdad, Iraq; b College of Medicine, University of Baghdad, Baghdad, Iraq; c Faculty of Medicine, Bahri University, Khartoum, Sudan; d Medical City, Teaching Labs National Center, Baghdad, Iraq.

**Keywords:** ANCA-associated vasculitis, granulomatosis with polyangiitis, necrotizing scleritis, pulmonary mass

## Abstract

**Rationale:**

Granulomatosis with polyangiitis (GPA) is a rare, life-threatening vasculitis characterized by necrotizing granulomatous inflammation. Isolated necrotizing anterior scleritis as the sole initial manifestation is distinctly rare. The presence of a clinically silent pulmonary mass without ear, nose, and throat or renal involvement makes this presentation exceptional and diagnostically challenging.

**Patient concerns:**

A 31-year-old man presented with recurrent unilateral necrotizing anterior scleritis resistant to corticosteroids and immunosuppressants, without systemic symptoms.

**Diagnoses:**

This single-patient case report, prepared in accordance with CAse REport guidelines, revealed mildly elevated anti-proteinase 3 antibodies (25 AU/mL) in laboratory tests. Chest computed tomography unexpectedly showed a 5-cm solitary pulmonary mass. Histopathology demonstrated necrotizing granulomatous inflammation with multinucleated giant cells and vasculitis, confirming GPA.

**Interventions:**

The patient was treated with pulsed intravenous methylprednisolone followed by monthly cyclophosphamide, then transitioned to rituximab maintenance therapy.

**Outcomes:**

He achieved marked improvement in ocular inflammation and visual function, with no new systemic manifestations during follow-up.

**Lessons:**

This case illustrates a rare diagnostic pathway in which isolated, treatment-resistant necrotizing scleritis led to the detection of clinically silent pulmonary GPA. It highlights the importance of considering systemic vasculitis in atypical or refractory scleritis and underscores the critical role of tissue biopsy when serologic findings are mild or nonspecific. Furthermore, this first documented Middle Eastern case adds educational value for ophthalmologists and rheumatologists, illustrating that early multidisciplinary evaluation and timely rituximab therapy can prevent irreversible ocular damage and systemic progression.

## 1. Introduction

Granulomatosis with polyangiitis (GPA), formerly known as Wegener granulomatosis, is a rare, potentially life-threatening autoimmune vasculitis characterized by necrotizing granulomatous inflammation predominantly affecting small- to medium-sized vessels. From an immunopathogenic standpoint, GPA involves complex cytokine-mediated inflammation. Elevated levels of interleukin-6, interleukin-17, tumor necrosis factor-alpha, and other chemokines promote neutrophil activation, endothelial injury, and granuloma formation.^[1–3]^ Recent transcriptomic and signaling studies have highlighted the role of intracellular cascades such as mitogen-activated protein kinase (MAPK) and mechanistic target of rapamycin in amplifying these inflammatory responses.^[4,5]^ MAPK pathways, including extracellular signal-regulated kinase, c-Jun N-terminal kinase, and p38, modulate cytokine release and neutrophil priming, while crosstalk with mechanistic target of rapamycin signaling contributes to persistent vascular inflammation.^[6,7]^ These insights help explain the severe tissue-destructive and granulomatous features characteristic of GPA.

GPA is classically affects the upper and lower respiratory tracts and kidneys, although virtually any organ system can be involved. Ocular manifestations are relatively common in GPA, with estimates suggesting that up to 50% of patients experience some form of eye involvement during the course of their disease.^[8]^

Among the ocular presentations, scleritis is a particularly severe and vision-threatening condition. It may be diffuse, nodular, or necrotizing, with the latter carrying a higher risk of globe perforation and permanent vision loss.^[9]^ Isolated necrotizing scleritis as the sole initial presentation is distinctly rare, reported in <10% of patients at onset.^[8,9]^

Recognizing scleritis as a potential sign of GPA is critical for early diagnosis and treatment, as delayed recognition can result in irreversible ocular damage and progression to systemic organ involvement.^[8,9]^

Recent studies have reported cases where GPA presented with isolated ocular symptoms. For instance, a 2024 case described a 25-year-old woman whose GPA was diagnosed after presenting with a single ocular lesion, emphasizing the diagnostic challenge of isolated ocular involvement.^[10]^ Another 2024 report discussed a 40-year-old female who initially presented with biparietal headache, unresponsive to medication, which then led to ophthalmoplegia, highlighting the diverse presentations of GPA.^[11]^ Additionally, a 2025 study detailed a rare case of presumed limited GPA with sectoral scleritis and ciliary body granuloma that exhibited resistance to treatment but was eventually controlled with rituximab, mycophenolate mofetil, and dexamethasone implant.^[12]^

These cases underscore the importance of considering GPA in patients with isolated ocular symptoms. However, to our knowledge, no previous case from the Middle East has documented such a sequence – isolated scleritis preceding systemic disease by nearly 2 years, confirmed by lung histopathology and successfully treated with rituximab. This progression is uncommon and highlights the importance of maintaining a high index of suspicion for systemic vasculitis in patients with severe or atypical scleritis.

Our case contributes to the existing literature by presenting a unique sequence of events: an isolated anterior manifestation as the initial presentation of GPA, progressing to a silent lung mass before diagnosis. This case highlights the importance of considering GPA in the differential diagnosis of patients presenting with isolated ocular symptoms, even in the absence of systemic signs, as early recognition and evaluation can enable timely treatment and prevent irreversible ocular or systemic damage.

## 2. Case presentation

A 31-year-old previously healthy Iraqi male presented with a 2-year history of recurrent, severe left eye pain and redness. The pain was deep, rated 8/10 on the visual analog scale^[13]^ occasionally waking him from sleep, and significantly affecting daily activities. He reported no other systemic symptoms.

In August 2022, he developed generalized fatigue, night sweats, and joint pain and was diagnosed with seropositive rheumatoid arthritis (RA) based on positive rheumatoid factor and clinical assessment. He was started on hydroxychloroquine and sulfasalazine with partial symptom control.

By February 2023, ocular symptoms first appeared as he developed painful redness in his left eye. He was diagnosed with scleritis, presumed secondary to RA, and hydroxychloroquine was discontinued. Topical corticosteroids were prescribed, but the symptoms persisted. Over the following months, he was treated with oral prednisolone, azathioprine, and deflazacort, though adherence was inconsistent and disease control was suboptimal.

In October 2024, due to worsening visual loss, he was referred to the Ophthalmology and Rheumatology departments at Baghdad Teaching Hospital. Ocular examination showed marked conjunctival injection, scleral thinning, and significantly reduced visual acuity in the left eye (counting fingers). The diagnosis of GPA was confirmed following a lung biopsy. Anti-proteinase 3 (anti-PR3) antibody was mildly positive at 25 AU/mL (reference < 20). Ocular ultrasound confirmed initial anterior scleritis progressively evolved into posterior necrotizing scleritis with associated retinal detachment.

Laboratory investigations showed leukocytosis (white blood cells 17 × 10^9^/L), neutrophilia (80%), thrombocytosis (450 × 10^9^/L), and elevated rheumatoid factor (55 IU/mL). Antinuclear antibody, anti-cyclic citrullinated peptide, anti-double-stranded deoxyribonucleic acid, and human leukocyte antigen-B27 were negative. Anti-PR3 antibodies were mildly positive (25 AU/mL). Other infectious workups and tumor markers were unremarkable.

Infectious screening, including blood cultures, hepatitis B and C serologies, human immunodeficiency virus, syphilis, and Quantiferon-TB Gold (QIAGEN), were all negative. Inflammatory markers were elevated (C-reactive protein 85 mg/L; erythrocyte sedimentation rate 72 mm/h).

As part of a systemic evaluation, a contrast-enhanced chest computed tomography unexpectedly revealed a 5-cm solitary, well-defined mass in the left lower lobe (Fig. 1) and a smaller right nodule of <1 cm; both lesions were enhanced after contrast.

**Figure 1.**
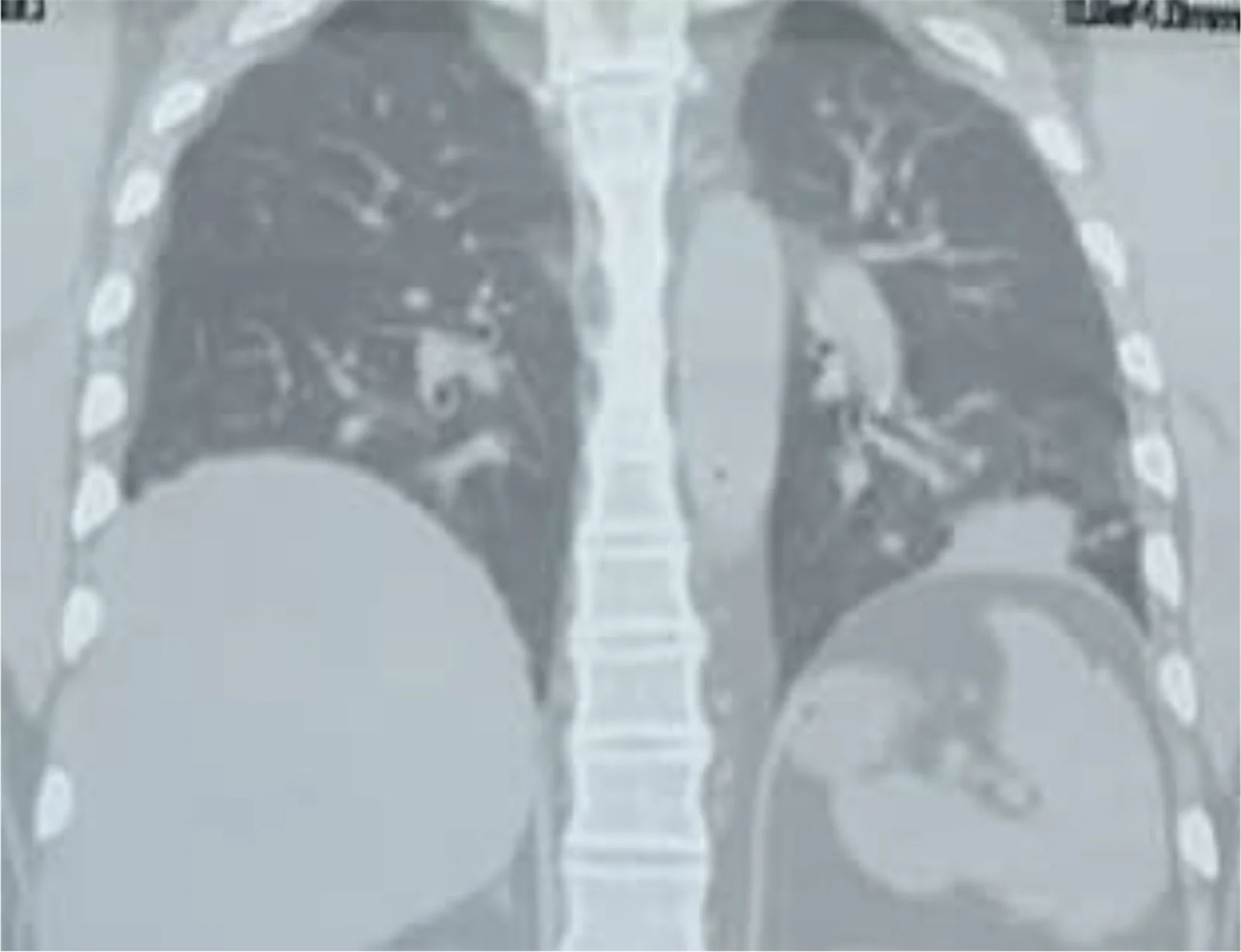
Contrast-enhanced chest CT showing a well-defined 5-cm mass in the left lower lobe. CT = computed tomography.

Given the patient’s age, smoking status, and isolated pulmonary finding, malignancy was initially suspected. The patient was referred to the thoracic surgery team, and video-assisted thoracoscopic surgery with mass removal was advised. Due to intraoperative difficulty, the approach was converted to an open thoracotomy via a left subcostal incision, and the left lower lobe was completely excised and sent for histopathological examination.

Histopathology revealed necrotizing granulomatous inflammation characterized by basophilic, suppurative necrosis with abundant neutrophils, karyorrhexis, and multinucleated giant cells. Medium- and small-sized arteries and veins showed inflammation with neutrophilic, lymphocytic, and histiocytic infiltrates, features consistent with GPA. This histologic confirmation, in the context of necrotizing scleritis and positive anti-PR3 serology, led to a definitive diagnosis of GPA.

After histopathologic confirmation of GPA, systemic therapy was initiated with prednisolone 60 mg/d, gradually tapered over subsequent months, and rituximab 1 g intravenously on day 0 and day 15, followed by maintenance infusions every 6 months. Methotrexate 15 mg/wk with folic acid 5 mg/d, omeprazole 40 mg/d, vitamin D3, and calcium supplements were prescribed for maintenance and supportive care. This regimen was chosen in accordance with European Alliance of Associations for Rheumatology recommendations for antineutrophil cytoplasmic antibodies-associated vasculitis management (Fig. 2).^[14]^

**Figure 2.**
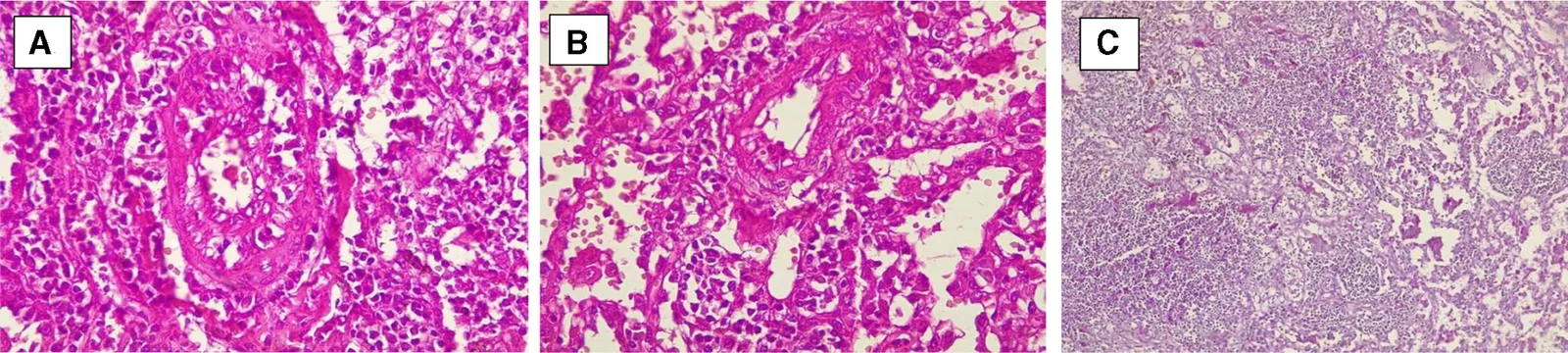
Histopathological slides stained with hematoxylin and eosin at 200× (A) and 400× (B) magnification, showing necrotizing granulomatous inflammation with multinucleated giant cells. (C) Periodic acid–Schiff stain at 200× demonstrates necrotizing vasculitis and granulomatous inflammation, negative for fungal organisms, supporting the diagnosis of granulomatosis with polyangiitis.

The patient remains under close follow-up by the Rheumatology and Ophthalmology Departments at Baghdad Teaching Hospital for ongoing management and monitoring.

At 6-month follow-up, the patient remained in clinical remission with resolution of ocular inflammation and no new systemic involvement. He continues regular follow-up for maintenance therapy and monitoring.

## 3. Discussion

Scleritis is an uncommon initial manifestation of GPA, though ocular involvement is reported in up to 50% of GPA patients over the course of the disease. In particular, necrotizing or nodular anterior/posterior scleritis may represent an early clue to systemic vasculitis, particularly when it is unilateral, painful, and refractory to standard anti-inflammatory therapy.^[9,15]^

The predominance of necrotizing granulomatous inflammation with abundant neutrophils, multinucleated giant cells, and vascular infiltrates in our patient is consistent with the known inflammatory processes described in GPA and illustrates how localized disease may precede overt systemic involvement. In line with recent literature, MAPK signaling plays a central role in mediating immune and inflammatory responses, highlighting the intracellular mechanisms that contribute to tissue injury in GPA.^[16]^

Our case underscores several key clinical challenges. The patient presented initially with isolated unilateral nodular scleritis, a finding that may be easily misattributed to idiopathic or autoimmune etiologies, such as RA. A review by Dammacco et al (2023) reported that over one-third of their GPA patients had ocular involvement, with presentations ranging from scleritis and episcleritis to orbital disease, underscoring the variability of ocular presentations.^[17]^

In our case, the lack of initial systemic signs delayed the suspicion of GPA. Recent literature supports the understanding that GPA usually presents with systemic manifestations early in the disease course, with ocular involvement often appearing later or in conjunction with other systemic symptoms.^[18]^ However, persistent pain, scleral thinning, and recurrence despite conventional therapy raised concern for vasculitic etiology. Notably, GPA was diagnosed only after lung mass biopsy revealed necrotizing granulomatous inflammation with multinucleated giant cells.

Compared to previously reported cases, this case is distinguished by the delay between ocular presentation and systemic diagnosis, as well as the initial lack of renal involvement. Several case series note that in many patients with GPA-associated scleritis, the disease either presents concurrently with other organ involvement or is diagnosed following characteristic sinus or pulmonary findings.^[15,19,20]^ In contrast, our case featured scleritis as the initial and primary symptom, with other features only emerging later. This atypical sequence highlights the importance of not dismissing persistent or recurrent scleritis, especially nodular or necrotizing forms, as benign.

Management of GPA with ocular involvement typically requires systemic immunosuppression. Corticosteroids alone may achieve transient control, but cyclophosphamide or rituximab are often necessary to achieve remission.^[19,21]^ In our patient, the combination of pulsed intravenous methylprednisolone and monthly cyclophosphamide induced significant improvement, consistent with treatment recommendations from previous series.

Importantly, the case illustrates the diagnostic delay that can occur when scleritis is initially managed in isolation. Other reports have shown that biopsy of scleral or pulmonary lesions may be required in cases where antineutrophil cytoplasmic antibodies is negative or inconclusive.^[22,23]^ In our case, although chest imaging suggested pulmonary involvement, the definitive diagnosis of GPA was confirmed only after histopathological examination of a biopsied lung mass. This highlights the importance of tissue biopsy, especially in atypical or limited presentations where serologic markers alone may not be conclusive. The absence of glomerulonephritis at presentation is also notable; although renal involvement is eventually seen in most cases of GPA, some patients, like ours, present with limited disease.^[19]^

Ultimately, this case underscores the importance of early systemic evaluation in patients with nonresponsive or atypical scleritis. A multidisciplinary approach involving ophthalmology, rheumatology, and pulmonology enabled timely diagnosis and appropriate immunosuppressive treatment, which preserved vision and prevented further systemic deterioration. Long-term follow-up beyond 6 months is required to confirm sustained remission and exclude delayed systemic relapse.

In summary, this case demonstrates that GPA can initially present with isolated ocular inflammation, underscoring the need for vigilance in atypical scleritis cases. Prompt systemic evaluation, including tissue biopsy when indicated, is essential to avoid delays in diagnosis and treatment. Further research into the cellular mechanisms of localized ocular disease and optimized diagnostic pathways could improve early recognition and outcomes in patients with unusual GPA presentations. In patients with severe or refractory scleritis, systemic vasculitis should be considered even in the absence of ear, nose, and throat or renal involvement. Despite the single-case design, this report provides valuable clinical and histopathological insights that can inform the evaluation and management of similar atypical presentations.

## 4. Conclusion

This case highlights the diagnostic complexity of GPA when presenting solely with ocular symptoms. Anterior or posterior necrotizing scleritis, particularly when unilateral, persistent, and unresponsive to conventional therapy, should raise suspicion for an underlying systemic vasculitis. Our patient’s delayed diagnosis reflects the challenges clinicians face when systemic manifestations are absent or minimal at onset. Crucially, GPA was only confirmed following histopathological examination of a pulmonary lesion, emphasizing the value of tissue diagnosis in atypical presentations.

Early recognition and systemic evaluation are vital to prevent irreversible ocular damage and systemic complications. This case reinforces the need for a high index of suspicion and multidisciplinary collaboration when managing scleritis of unclear etiology. Clinicians should remain alert to the possibility of GPA in similar scenarios to avoid diagnostic delay and improve patient outcomes.

## Author contributions

**Conceptualization:** Nabaa Ihsan Awadh, Marafi Jammaa Ahmed.

**Data curation:** Ahmed Dheyaa Al-Obaidi, Karam Fareed Nawara.

**Investigation:** Nabaa Ihsan Awadh.

**Methodology:** Faiq I. Gorial, Mohammed Alghannami, Karam Fareed Nawara, Marafi Jammaa Ahmed, Aya Ahmed Shimal.

**Project administration:** Faiq I. Gorial.

**Resources:** Faiq I. Gorial, Mohammed Alghannami.

**Supervision:** Ibtisam Hussein Al Obaidi.

**Validation:** Aya Ahmed Shimal, Ibtisam Hussein Al Obaidi.

**Visualization:** Ahmed Dheyaa Al-Obaidi.

**Writing – original draft:** Ahmed Dheyaa Al-Obaidi, Mohammed Alghannami, Karam Fareed Nawara, Marafi Jammaa Ahmed.

**Writing – review & editing:** Nabaa Ihsan Awadh, Marafi Jammaa Ahmed, Aya Ahmed Shimal, Ibtisam Hussein Al Obaidi.
